# Champions for improved adherence to guidelines in long-term care homes: a systematic review

**DOI:** 10.1186/s43058-021-00185-y

**Published:** 2021-08-03

**Authors:** Amanda M. Hall, Gerd M. Flodgren, Helen L. Richmond, Sheila Welsh, Jacqueline Y. Thompson, Bradley M. Furlong, Andrea Sherriff

**Affiliations:** 1 Primary Healthcare Research Unit (PHRU), Faculty of Medicine, Memorial University of Newfoundland, Janeway Hostel, Health Sciences Centre, 300 Prince Philip Parkway, St. John’s, NL A1B 3 V6 Canada; 2 Division for Health Services, Norwegian Institute of Public Health, Marcus Thranes gate 6, Oslo, 0403 Norway; 3 Public Health Department, NHS Ayrshire & Arran, Ayr, UK; 4 Institute of Inflammation and Ageing, University of Birmingham, Birmingham, UK; 5 School of Medicine, Dentistry and Nursing, College of MVLS, University of Glasgow, Glasgow, UK

**Keywords:** Champion, Implementation, Long-term care, Evidence-based care, Guidelines

## Abstract

**Background:**

The champion model is increasingly being adopted to improve uptake of guideline-based care in long-term care (LTC). Studies suggest that an on-site champion may improve the quality of care residents’ health outcomes. This review assessed the effectiveness of the champion on staff adherence to guidelines and subsequent resident outcomes in LTC homes.

**Method:**

This was a systematic review and meta-analyses of randomised controlled trials. Eligible studies included residents aged 65 or over and nursing staff in LTC homes where there was a stand-alone or multi-component intervention that used a champion to improve staff adherence to guidelines and resident outcomes. The measured outcomes included staff adherence to guidelines, resident health outcomes, quality of life, adverse events, satisfaction with care, or resource use. Study quality was assessed with the Cochrane Risk of Bias tool; evidence certainty was assessed using the GRADE approach.

**Results:**

After screening 4367 citations, we identified 12 articles that included the results of 1 RCT and 11 cluster-RCTs. All included papers evaluated the effects of a champion as part of a multicomponent intervention. We found low certainty evidence that champions as part of multicomponent interventions may improve staff adherence to guidelines. Effect sizes varied in magnitude across studies including unadjusted risk differences (RD) of 4.1% [95% CI: − 3%, 9%] to 44.8% [95% CI: 32%, 61%] for improving pressure ulcer prevention in a bed and a chair, respectively, RD of 44% [95% CI: 17%, 71%] for improving depression identification and RD of 21% [95% CI: 12%, 30%] for improving function-focused care to residents.

**Conclusion:**

Champions may improve staff adherence to evidence-based guidelines in LTC homes. However, methodological issues and poor reporting creates uncertainty around these findings. It is premature to recommend the widespread use of champions to improve uptake of guideline-based care in LTC without further study of the champion role and its impact on cost.

**Trial registration:**

PROSPERO CRD42019145579. Registered on 20 August 2019.

**Supplementary Information:**

The online version contains supplementary material available at 10.1186/s43058-021-00185-y.

Contributions to the literature

- This is the first systematic review assessing the effect of a champion intervention for improving adherence to guideline-based care.
- Our review shows that champion interventions are promising for improving adherence to evidence-based care in long-term care (LTC) settings but that further study is required before widespread use can be recommended.
- Future work in this area requires the use of more robust methods and should evaluate the cost-effectiveness of the champion, assess staff adherence directly and report findings according to accepted high-quality reporting standards (e.g. CONSORT statement).

## Background

Despite its benefits, care provided to residents in long-term care (LTC) homes (e.g. oral hygiene care, pressure ulcer prevention and infection control) is not always evidence-based [1, 2]. This is in part due to the changing needs of older residents in LTC home settings (e.g. decreased ability to perform self-care and/or physical activities of daily living) resulting in the need for increasing staff education and policies about the best methods to provide this care for residents. To this end, guidelines and interventions have been developed for many problem topic areas facing LTC homes. These include, for example, guidelines for the prevention and treatment of pressure ulcers [3, 4], oral health care guidelines [5] and interventions designed to improve well-being for patients with dementia [4] or to reduce functional decline [6]. However, even with the increase of toolkits and training to assist with uptake of best practices, implementation of these practices has been sub-optimal, possibly due to multiple factors ranging from staff turnover and competing interests to forgetfulness [7]. Adherence to the guidelines or intervention protocol must be also be considered in terms of implementation outcomes since the extent to which guidelines or interventions work is directly impacted by whether or not they were implemented as intended [8].

The champion model is being increasingly adopted in areas of care that have proven resistant to improvement, e.g. oral health [9], incontinence [10] and infection control [11]. Studies suggest that having at least one on-site champion may help improve the quality of care in that area and thereby the residents’ health outcomes [11–16]. Although there is no standard definition of a champion in the implementation literature, common elements of a champion for supporting change in healthcare settings include being a staff member (who either volunteers or is assigned an additional level of responsibility), who may perform a number of different roles in order to improve staff adherence to a particular guideline, policy or intervention [17]. A champion is different than an opinion leader [18]; unlike opinion leaders, champions are typically equal to their peers or colleagues and do not have a higher social or work status [18]. Champions may fill a diverse number or combination of roles such as advocating and/or leading practice change [19, 20], building relationships and educating peers and other staff to encourage and engage them in QI initiatives [19, 21] and acting as a resource or mentoring (including modelling and reinforcing desired behaviour) to facilitate the implementation of protocol interventions [19, 22].

To date, there has been no review of the effectiveness of the champion model for improving adherence to guideline-based care in LTC homes. This systematic review assessed the effectiveness of the champion on staff adherence to guidelines and subsequent resident outcomes in LTC homes.

## Methods

Here, we provide a succinct overview of our methods, a thorough description of which is included in our prospectively registered protocol (PROSPERO 2019 CRD42019145579). We developed the protocol in accordance with guidance from the Cochrane Effective Practice and Organization of Care (EPOC) group [23] and the Preferred Reporting Items for Systematic Reviews and Meta-Analyses (PRISMA) [24] (Additional file 1).

### Searches

We searched four databases (CENTRAL, MEDLINE, Embase, CINAHL), two trial registries (ICTRP and ClinicalTrials.gov) and three sources of grey literature (ProQuest Dissertations and Theses, Science Citation Index Expanded, and Conference Proceedings Citation Index - ISI Web of Knowledge) from inception to July 2020 (as well as reference lists of included studies and relevant reviews). We used a sensitive search strategy with terms for champions, long-term care homes and older adults (Additional file 2).

### Study characteristics

We used the Population, Intervention, Comparator, Outcomes (PICO) framework [25] to define our selection criteria.

#### Population

We included studies with participants aged 65 years and older located in LTC homes where the intervention involved designating a nursing home staff member as a champion. The staff member could include registered nurses, licensed practical nurses, personal care attendants, personal support workers or nursing aides.

#### Intervention

We defined a champion as an internal nursing staff member who had an implementation-related role, had received supplementary training, assumed responsibility for a specific topic area (e.g. pressure ulcer prevention) and may have acted as a key contact person with external healthcare providers (e.g. dieticians, physiotherapists, oral health specialists). Importantly, we excluded studies where the designated champion was filled by an external, high-level, educationally-influential opinion leader such as those described in Flodgren et al. [18]. Guided by the Institute of Medicine’s definition of guidelines, we included any intervention that aimed to implement a clinical practice guideline or an evidence-based recommendation that optimised patient care. Moving forward, we will use the term “guidelines” to refer to both clinical practice guidelines and evidence-based recommendations as described above.

#### Comparator(s)

We included the following comparison groups:

1. No intervention group (no implementation strategies tested)
2. Another intervention (which may or may not have included a champion)

#### Outcomes

We selected outcomes for this review from the list recommended by the Effective Practice and Organization (EPOC) group [26]. The primary outcome of this study was adherence to guidelines (a quality-of-care outcome outlined by the EPOC group [26]. Secondary outcomes included other EPOC-recommended outcomes such as patient outcomes (resident health outcomes, quality of life, satisfaction with care, adverse events) and resource use.

#### Study designs

We included only randomised controlled trials (RCTs) and cluster RCTs, as these are considered the gold-standard study design to assess the effectiveness of an intervention.

### Study selection and data extraction

Titles, abstracts and full texts were independently screened by two authors in Covidence to identify RCTs that met the inclusion criteria [27]. We extracted information about the study characteristics, interventions, and outcomes [28]. Disagreements were resolved through discussion and, where necessary, adjudicated by a third author. When required, we contacted authors of studies to obtain data not available in the publication.

### Quality assessment of included studies

Two authors independently assessed risk of bias (RoB) using the 9-item Cochrane risk of bias tool [29]. We considered three of the Cochrane RoB items to be essential (random sequence generation, allocation concealment, and incomplete outcome data). If we found a study to have high or unclear RoB for any of these three items, we considered it at a high risk of bias [29].

### Contrasts

We assessed the following five comparisons: the effect of the champion as a stand-alone intervention compared to (i) no intervention or (ii) another intervention; (iii) the effect of the champion as part of an intervention compared to the same intervention without the champion (i.e., the additive effect of a champion); and the effect of the champion as part of a multicomponent intervention compared to (iv) no intervention or (v) another intervention.

### Data coding and synthesis

We categorised the level of involvement of the champion in the interventions using the following descriptions defined by the review team:

(i) Minor: Acted as role model and source of information for staff and possibly as a reminder of the intervention but was not responsible for educating staff or enacting any of the intervention components.
(ii) Moderate: In addition to the responsibilities of the minor role, helped the research team to educate or mentor staff or assisted other members of the research team with activities.
(iii) Major: In addition to the responsibilities of the moderate role, independently (i.e., without the research team) educated or mentored staff and enacted other components of the intervention such as action planning or using new clinical tools at the site.

For the effectiveness analysis, we pooled the results of studies with sufficient homogeneity of participants, interventions and outcomes and acceptable statistical heterogeneity (*i*^2^ < 50%) [30]. Given that the majority of our studies were cluster-RCTs, we used the adjusted between-group difference where possible, adjusted risk difference (RD) for dichotomous outcomes and adjusted mean difference (MD) for continuous outcomes. For cluster RCTs that adjusted for clustering in their analysis and reported the adjusted between-group difference, this score was used in the meta-analysis [31]. We used a conservative random-effects model for all meta-analyses using the generic inverse variance outcome method to allow for pooling of adjusted between-group differences [32]. If it was not possible to pool the results across studies due to heterogeneity, we reported a qualitative assessment of the effect [33].

Two review authors independently determined the certainty of the evidence for each outcome (high, moderate, low and very low) using the five GRADE considerations [34]. We produced a GRADE summary of findings table for each comparison [29, 35, 36].

## Results

### Results of the search

**Fig. 1:**
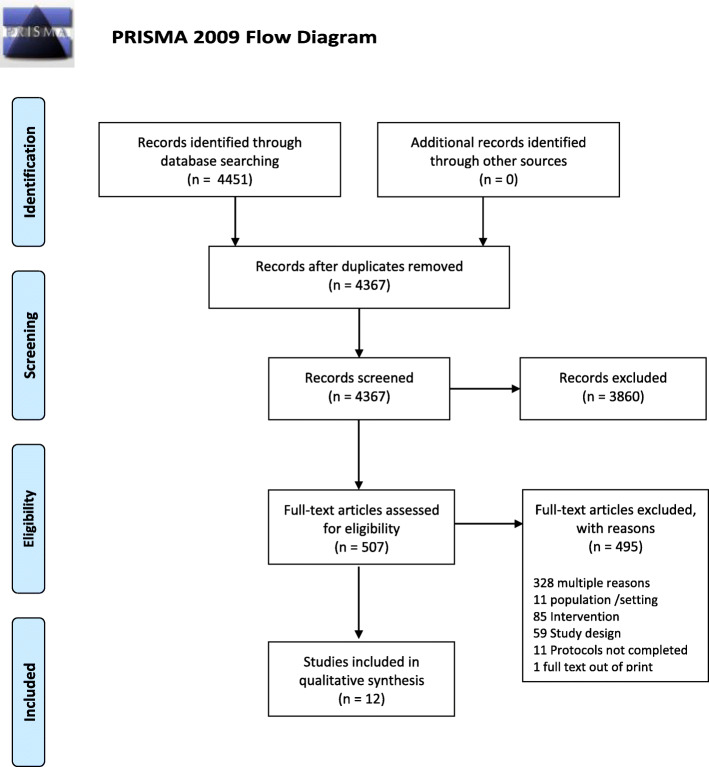
PRISMA flow diagram of the systematic literature search

Electronic database searches identified 4367 unique citations (Fig. 1), 3860 of which were excluded following title and abstract screening. We reviewed 507 full texts; 328 were irrelevant and an additional 167 studies were excluded with reasons. Eleven cluster RCTs and one RCT were therefore included in the review [5, 6, 37–46].

#### Description of included studies (Table 1)

The included studies were conducted in Australia [37, 38], Belgium [5, 39], Canada [40], France [41], the Netherlands [42, 43], the UK [44, 45] and the USA [6]. There was also one multi-country study [46]. Ten studies targeted nursing staff, five of which also targeted additional care staff including physicians or allied health professionals (e.g. physiotherapists, pharmacists) [5, 38, 39, 41, 45] and two studies targeted nursing aides exclusively [6, 40]. The behaviours targeted by the interventions included adherence to guidelines for provision of oral hygiene [5, 40, 42], dementia care [43, 44], function-focused care [6] and palliative care [46], as well as assessment and management of malnutrition [37], detection of delirium [45], detection of depression [38], prevention of pressure ulcers [39] or infections [41].

**Table 1:** Study characteristics of included studies

Study, Year, Country	Design	Target Behaviour—implementation of:	Comparison	Intervention	Outcomes Assessed					Risk of bias
					Staff adherence	Resident clinical outcome	Resident QoL	Adverse events	Resource use	
Beekman 2013 [47] **Belgium**	Cluster-randomised controlled trial	A pressure ulcer clinical decision-making support system	No intervention+ education	Champion + decision support + education + monitoring and feedback + reminders	Y	Y	N	N	N	H
Chami 2012 **France** [41]	Cluster-randomised controlled trial	A hygiene encouragement programme to reduce infection rates	No intervention	Champion + education + reminders + resources	N	Y	N	N	N	H
DeVisschere 2012 **Bekgium** **[**5**]**	Cluster-randomised controlled trial	An oral hygiene guideline	No intervention	Champion + education + oral health care products	N	Y	N	N	N	H
Gaskill 2009 **Australia** [37]	Cluster-randomised controlled trial	A malnutrition risk assessment and strategies to reduce malnutrition levels	No intervention+ Posters	Champion + education + reminders + risk assessment tools	N	Y	N	N	N	H
Livingston 2019 **UK** [44]	Cluster-randomised controlled trial	Managing agitation and raising quality of life in people with dementia	No intervention	Champion + Education + role play/practice + progress monitoring forms	N	Y	Y	N	Y	H
MacEntee 2007 **Canada** **[**40]	Cluster-randomised controlled trial	An oral health-related education programme	No intervention+ education	Champion + education + nurse educator	N	Y	N	N	N	H
McCabe 2013 **Australia** [38]	Randomised controlled trial	A depression recognition programme	No intervention	Arm 1: Training Arm 2: Champion + training	Y	N	N	N	N	H
Resnick 2011 **USA** [6]	Cluster-randomised controlled trial	A protocol to reduce functional decline	No intervention+ education	Champion + education + goal setting + mentoring and motivation	Y	Y	N	Y	Y	H
Siddiqi 2016 **UK** [45]	Cluster-randomised controlled trial	A protocol for delirium prevention and management	No intervention	Champion + education	Y	Y	Y	Y	Y	H
Van den Block 2019 **Multiple**	Cluster-randomised controlled trial	A programme to incorporate nonspecialist palliative care in nursing homes	No intervention	Champion + train-the-trainer model	N	Y	N	N	N	H
Van der Putten 2013 **Netherlands** [42]	Cluster-randomised controlled trial	An oral health programme	No intervention	Champion + train-the-trainer model	N	Y	N	N	N	H
Van de Ven 2013 **Netherlands** [43]	Cluster-randomised controlled trial	A dementia care mapping guideline to identify triggers of well/ill-being of residents	No intervention	Champion + education + audit and feedback + action plans	N	Y	Y	N	N	H

#### Description of the champion intervention (Table 2; Additional file 3)

All included studies evaluated the effects of a champion as part of a multicomponent intervention. Where reported, the duration of the intervention ranged from 4 to 16 months. The frequency of how often the different intervention components were administered was poorly reported.

**Table 2:** Intervention details of included studies

Category	Champion duties Note: these duties are beyond those listed in our inclusion criteria which are: received additional training in a topic area, had some responsibility for implementing recommendations in that topic area, generally act as a role model and/or key contact for staff about the topic area										Other implementation strategies not delivered by champion Note: if components such as a training session for feedback were provided but only provided by the champion they have been ticked in the champion duty section and have not been ticked again in this section.										
Strategy	Liaison with researchers	Implementing new decision support/screening tool	Delivered staff training with or without practice	Action planning and/or goal setting	Mentoring/motivating of other staff	Monitoring performance	Delivering feedback to staff	Part of a champion team	Educational training session	Practice/model behaviour	Audit/monitoring	Feedback	Decision support/screening tools	Local mentoring/motivation	Reminders	Goal setting	Action plans	Monetary or other incentives	Additional objects/products to facilitate behaviour	Additional expert support	Depth of champion role in the project (major, moderate, minor)
**Study**																					
Beekman 2013 [47]	x		x				x		x		x	x	x		x				x		Moderate
Chami 2012 [41]	x		x								x				x				x		Major
De Visschere 2012 [5]	x		x		x			x	x	x	x	x							x	x	Major
Gaskill 2009 [37]	x		x	x									x		x						Major
Livingston 2019 [44]	x							x	x		x	x		x			x			x	Minor
MacEntee 2007 [40]	x		x																		Major
McCabe 2013 [38]		x			x				x					x							Major
Resnick 2011 [6]	x			x	x				x		x			x		x		x			Major
Siddiqi 2016 [45]	x		x		x				x										x	x	Major
Van den Block 2020 [46]	x		x						x												Major
Van der Putten 2013 [42]	x		x		x				x		x	x							x		Major
Van de Ven 2013 [43]	x		x	x		x	x	x	x								x				Major

##### Training of and duties performed by the champion

In eight studies, a single staff member was appointed as a champion, while in four studies, a team of two or more champions was appointed [5, 43, 44, 46]. Details on how the champions were appointed were not provided. Training intensity (e.g. frequency, number and length of sessions) was only reported in two studies, which ranged from 2.5–15 h [6, 45].

In addition to receiving training and providing general oversight regarding the implementation of the recommendations, some champions were tasked with extra responsibilities. Most commonly, this included delivery of some or all of the education sessions to LTC home staff [5, 37, 39–43, 45, 46] and liaising with the research team from one-off sessions to develop an initial action plan to weekly sessions for implementation support [5, 6, 37, 39–46]. All additional duties are outlined in Table 2. Overall, we found the champion to play a major role in ten studies as they were responsible for enacting the majority of the intervention components [5, 6, 37, 38, 40–43, 45, 46]. In the remaining two studies, the champions had either a moderate [39] or minor role [44].

##### Other intervention components (affecting all LTC staff, including the champion)

All studies included education or training sessions for LTC home staff as one of the main intervention components. One study did not use any additional components beyond education [40, 46]. Amongst the 11 remaining studies, six also provided some form of mentoring or motivation training [5, 6, 38, 42, 44, 45], seven included monitoring via direct observation [5, 6, 39, 41–44], five provided written or oral feedback on performance [5, 39, 42–44], three used goal setting or action planning [6, 37, 43], three included staff reminders via posters [37, 39, 41] and one used pocket cards [39]. Six studies provided tools to help enact the desired behaviour [5, 38, 39, 41, 42, 45]. These included new screening tools to identify depression [38], delirium [45] and people at risk of pressure ulcers [39], as well as tools to improve the use of hand sanitiser [41] and oral hygiene products (for use with residents) [5, 42].

### Risk of bias in included studies (Table 1; Additional file 4)

We found that all studies were at high risk of bias. Amongst the three pre-specified criteria (appropriate sequence generation, concealed allocation and complete outcome data), most were judged to have an unclear risk of bias on randomisation (*n* = 5) and/or allocation (*n* = 10) due to lack of information to make an accurate judgement. In addition, more than half of the studies (*n* = 7) were found to have incomplete outcome data on the primary outcome of staff adherence and/or the resident outcomes. Also, 4 of the 11 cluster RCTs did not adjust for clustering in their analysis placing them at risk of presenting misleading results.

### Effectiveness of the champion interventions

We found no studies assessing the effect of a champion as a stand-alone intervention compared to no intervention or another intervention

### Effect of an intervention with a champion compared to the same intervention without the champion (Table 3)

#### Staff adherence

One RCT (69 staff) with low certainty evidence suggested that adding a champion to an implementation intervention may improve adherence (RD = 23% [95% CI: 5%, 52%]) to correctly detecting depression amongst residents [38]. No other outcomes were assessed in this comparison.

**Table 3:** Summary of findings table for included studies

**Champion(s) as part of an intervention compared with the same intervention without the champion for implementing various guidelines/hospital protocols in long-term care (LTC) homes**			
Population: Nursing Staff; Settings: LTC Homes; Intervention: Champions as part of an implementation intervention; Comparison: the same implementation intervention without the champion			
**Staff outcomes**			
**Outcomes**	**Impacts (risk differences (RD)s or mean differences (MD) are reported where possible)**	**No studies, clusters (staff)**	**Certainty** (GRADE)**
Adherence to best-practice recommendations*	It is uncertain if champions as part of a multi-component intervention may improve adherence to the use of a depression screening tool (RD = 23% [95% CI: 5%, 52%]) as compared to the same intervention but without the champion.	1 RCT (69 staff)	⊕⊖⊖⊖ Very low^1,2,3^
**Champion(s) as part of a multicomponent implementation intervention compared with no intervention for implementing various guidelines/hospital protocols in LTC homes**			
Population: Nursing Staff), and residents > 65 years old; Settings: LTC homes; Intervention: Champions as part of multi-component implementation intervention; Comparison: no intervention			
**Outcomes**	**Impact (risk differences (RD)s or Mean differences (MD) are reported where possible)**	**No studies, clusters (Staff)**	**Certainty** (GRADE)**
**Staff outcomes**			
Adherence to guidelines*	Champions as part of multicomponent interventions may improve staff adherence to guidelines. Champions, as part of multicomponent interventions, may improve staff adherence to guidelines (pressure ulcer prevention, function-focused care, and depression identification). The effect sizes (unadjusted RD) ranged from 4.1% to 44% improvement across studies. Note: The effect unadjusted RDs varied in magnitude across studies: pressure ulcer prevention in a bed and a chair respectively (4.1% [95% CI: − 3%, 9%] to 44.8% [95% CI: 32%, 61%]), identifying depression (44% [95% CI: 17%, 71%]), providing function-focused care (21% [95% CI: 12%, 30%]).	2 CRCTs,1 RCT, 15 clusters (260 staff)	⊕⊕⊖⊖ Low^1,2^
**Resident outcomes**		**No studies, clusters (residents)**	**Certainty** (GRADE)**
**Oral hygiene**^**a**^ (pooled data)	Champions, as part of multicomponent interventions, possibly reduce the levels of dental plaque (adjusted MD = − 0.28 [95% CI: − 0.55, 0.00]; *n* =167) and denture plaque (adjusted MD = − 0.34 [95% CI: − 0.50, − 0.18]; *n* = 388). One study, that could not be included in the meta-analysis reported a reduction in oral debris (adjusted MD = − 0.2 [95% CI: − 7.3, 7.0]; *n* = 113).	3 CRCTs, 37 clusters (640 residents)	⊕⊕⊕⊖ Moderate^1^
**Agitation**^**b**^ (pooled data)	Champions, as part of multicomponent interventions, may have little or no effect on resident’s level of agitation (adjusted MD = 0.49 [95% CI: − 2.39, 3.37]).	2 CRCTs, 31 clusters (503 residents)	⊕⊕⊖⊖ Low^1,2^
**Other clinical outcomes**^**c**^	It is uncertain whether champions, as part of a multifaceted intervention may improve other clinical outcomes because the certainty of evidence is very low. Clinical Physical Function (unadjusted MD = 4.77 [95% CI: 1.39, 8.15]), Pressure ulcer prevalence (unadjusted RD = 0.00 [95% CI: − 0.03, 0.02]), Moderate-severe malnourishment (adjusted OR = 1.6 [95% CI: 0.8, 3.1])^h^, prevalence of delirium (unadjusted RD = − 0.03 [95% CI: − 0.10, 0.04]), infections (adjusted hazard ratio = 0.99 [95% CI: 0.87, 1.12])^h^, comfort in the last week of dying (adjusted MD = 0.91 [95% CI: − 1.03, 2.85]).	6 CRCTs, M:12.5 clusters (4–47)	⊕⊖⊖⊖ Very low^1,2,3^
**Adverse outcomes**^**d**^	It is uncertain whether champions, as part of a multifaceted interventions may have an effect on adverse outcomes because the certainty of evidence is very low. Unadjusted RDs for (i) injury (RD = 7%; [95% CI: − 5%, 20%]), (ii) falls (RD = 1%; [95% CI: − 14, 16%]) and (iii) ED visits related to falls (RD = 4%; [95% CI: − 2%, 10%]).	1 CRCT, study (4 clusters, 169 residents)	⊕⊖⊖⊖ Very low?^1,2,3^
**Quality of life**^**e**^ (pooled data)	It is uncertain whether champions, as part of multicomponent interventions may improve resident’s quality of life (unadjusted MD = 0.03 [95% CI: − 0.01, 0.07])	3 CRCTs, 45 clusters (653 residents)	⊕⊖⊖⊖ Very low?^1,2,3^
**Satisfaction with care**^**f**^	It is uncertain whether champions, as part of a multifaceted intervention may improve residents’ satisfaction with care because the certainty of evidence is very low. [adjusted MD 1.72; 95% CI: − 0.15; 3.59]	1 CRCT, 73 clusters (913 residents)	⊕⊖⊖⊖ Very low^1,2,3^
**Resource use**^**g**^ (hospital admissions)	It is uncertain whether champions as part of a multicomponent intervention may decrease the number of hospital admissions. Meta-analysis was not performed due to heterogeneity, unadjusted RD ranged from 7% [95% CI: − 15%, 0%] to 22% [95% CI: − 37%, − 7%] for those in the champion intervention group.	2 CRCT,18 clusters (261 residents)	⊕⊖⊖⊖ Very low^1,2,3^

*CRCT* cluster randomised trial, *M* median, *OR* odds ratio, *RCT* randomised controlled trial

*The post-intervention risk differences were adjusted for pre-intervention differences between the comparison groups, where pre values were available. One of the three studies did not report baselines values and did not report on baseline similarities; for this study the unadjusted risk difference is reported

^a^Dental plaque was measured by the Silness and Loe validated plaque index and denture plaque was measured by the Augsburger and Elahi Methylene Blue disclosing solution, oral debris was measured by the Geriatric Simplified Debris Index. ^b^Agitation was measured by the primary caregivers using the Cohen-Mansfield Agitation Inventory. ^c^The outcomes were: Physical function (measured by the Barthel Index), pressure ulcer prevalence (measured by skin observation and categorised according to the 2009 EPUAP/NPUAP classification system), malnourishment (measured by the research team using the Subjective Global Assessment (SGA) nutrition assessment tool), delirium (measured by trained research assistants using the Delirium Rating Scale-Revised-98), infections (measured by research staff using medical case notes and biologic/radiologic data if available), comfort in the last week of life (measured by staff using the End-of-Life in Dementia Scale Comfort Assessment while dying (EOLD-CAD) tool). ^d^Adverse outcomes (measured with number of injuries, falls, and emergency visits related to falls) and ^e^Quality of life (measured by the EQ5D). ^f^Resource (measured by number of hospital admissions). ^g^Satisfaction (measured from a relative’s perspective using the End of-Life in Dementia–Satisfaction with Care tool). ^h^A RD was unable to be calculated and therefore the estimate provided in the paper (e.g. OR or HR) was reported. ** GRADE Working Group grades of evidence

High = This research provides a very good indication of the likely effect. The likelihood that the effect will be substantially different is low. Moderate = This research provides a good indication of the likely effect. The likelihood that the effect will be substantially different is moderate. Low = This research provides some indication of the likely effect. However, the likelihood that it will be substantially different is high. Very low = This research does not provide a reliable indication of the likely effect. The likelihood that the effect will be substantially different is very high. Substantially different = a large enough difference that it might affect a decision

Downgraded due to risk of bias, ^2^imprecision, ^3^inconsistency. Note: outcomes with data from single studies were automatically downgraded due to imprecision and inconsistency

### Effects of champions as part of multicomponent interventions compared to no intervention (Table 3)

#### Staff adherence

Staff adherence was assessed objectively by members of the research team in three studies (2 clusters RCTs and 1 staff-randomised RCT including 15 clusters and a total of 260 staff). Heterogeneity in the type of guidelines assessed, target behaviour, and adherence measures used across studies meant that meta-analysis was inappropriate. Overall, we found low certainty evidence that champions as part of multicomponent interventions may improve staff adherence to guidelines. The effect sizes varied in magnitude across studies including unadjusted risk differences (RD) of 4.1% [95% CI: -3%, 9%] to 44.8% [95% CI: 32%, 61%] for improving pressure ulcer prevention in a bed and a chair respectively [39], an RD of 44% [95% CI: 17%, 71%] for improving depression identification [38] and an RD of 21% [95% CI: 12%, 30%] for improving function-focused care to residents [6]. All results were unadjusted for baseline differences.

#### Resident clinical health outcomes

**Fig. 2:**
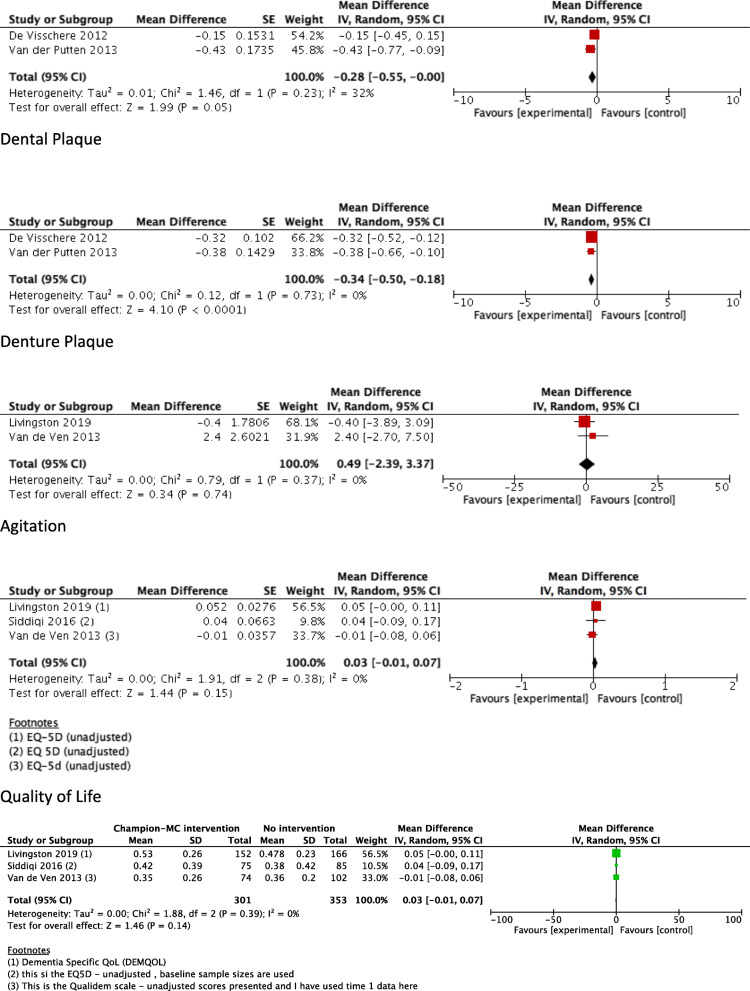
Meta-analyses comparing the effectiveness of a champion as part of a multicomponent intervention compared to no intervention on the following resident clinical health outcomes: dental plaque, denture plaque, agitation and quality of life

Eleven studies reported residents’ clinical health outcomes [5, 6, 37, 39–46]. Three assessed oral hygiene [5, 40, 42], two assessed agitation [43, 44] and the remaining five assessed either physical function [6], comfort in the last week of life [46], pressure ulcer prevalence [39], malnutrition [37], delirium [45] or infection rate [41]. Meta-analysis was not suitable for outcomes of oral hygiene and agitation (Fig. 2). We found moderate certainty evidence that residents in LTC homes with the champion intervention had slight reductions in dental plaque (adjusted MD = − 0.28 [95% CI: − 0.55, 0.00]; 37 clusters, 167 residents) and denture plaque (adjusted MD = − 0.34 [95% CI: − 0.50, − 0.18]; 37 clusters, 388 residents) and low certainty evidence of little or no effect of champion interventions on agitation levels (adjusted MD = 0.49 [95% CI: − 2.39, 3.37], 31 clusters, 503 residents). Amongst the other clinical outcomes, we found either no significant difference (malnutrition, comfort in the last week of life, delirium, infection rate, category II–IV pressure ulcer prevalence) or a slight improvement in the clinical outcome (physical function, category I–IV pressure ulcer prevalence) for those in the LTC facilities with the champion intervention. These results, however, were uncertain as they were based on very low certainty evidence from single studies (data presented in Table 3).

### Quality of life

Results from three studies (45 clusters, 653 residents) provide very low certainty evidence to suggest that champions as part of multicomponent interventions improve care for dementia and prevention of delirium, but have no effect on resident quality of life (unadjusted MD = 0.03 [95% CI: − 0.01, 0.07]).

### Adverse outcome

We found very low certainty evidence from one study (4 clusters, 169 residents) of no significant difference on resident adverse events related to a function-focused care programme between groups receiving the multicomponent intervention with a champion or no intervention. Unadjusted RDs for (i) injury (RD = 7% [95% CI: − 5%, 20%]), (ii) falls (RD = 1% [95% CI: − 14%, 16%]) and (iii) ED visits related to falls (RD = 4% [95% CI: − 2%, 10%]) [6].

### Satisfaction with care

We found very low certainty evidence from one study (73 clusters, 913 residents) that there is no significant difference in residents’ satisfaction with care between those receiving the champion intervention or no intervention (adjusted MD = 1.72 [95% CI: − 0.15, 3.59]) [46].

### Resource use

We found very low certainty evidence from two studies (18 clusters, 261 residents) of a reduction in hospital admissions for those groups receiving the champion as part of a multicomponent intervention. Meta-analysis was not performed due to differences in how hospital admissions were defined and timepoint assessed. Overall, the reductions reported as unadjusted RD ranged from 7% [95% CI: − 15%, 0%] [6] to 22% [95% CI: − 37%, − 7%] for those in the champion intervention group [6, 45].

## Discussion

### Summary of findings

This is the first systematic review assessing the effect of a champion intervention for improving adherence to guideline-based care in LTC homes. We found 12 RCTs testing a champion as part of a multicomponent intervention compared to no intervention. However, only three provided data on adherence; the majority instead assessed resident clinical health outcomes. Overall, our findings from the three studies in this comparison suggested that a champion as part of a multicomponent intervention may improve adherence to guidelines compared to no intervention. Importantly, since these interventions were multicomponent in nature, it is impossible to isolate the effectiveness of the champion from the other components. However, within each of these three studies, the champion played either a moderate or major role in the delivery of the intervention. For example, in all three studies, the champion delivered staff education/training and liaised with the research team to monitor progress and problem solve implementation issues as well as provide feedback to staff. Therefore, it is likely that they may have been a contributing factor to the effects on staff adherence. In addition, one of the three studies also assessed the effects of the intervention with and without a champion, which allowed us to estimate the additive effect of a champion [38]. The results of this study indicate that adherence to recommendations was greater when a champion was used, providing further support for the potential effectiveness of a champion as an implementation strategy. Taken together, we believe the evidence suggests that interventions that involve a champion and staff education and feedback on performance may improve staff adherence. However, given the moderate sample sizes of these three studies and the poor reporting of key risk of bias items, this estimate is considered to be of low to moderate certainty. Moreover, while we found one study that isolated the role of the champion and found it to be an effective strategy, this result is also very low certainty and needs further study.

With the exception of oral hygiene, there was either no significant difference or a slight improvement on resident outcomes for LTC homes with the champion intervention. For oral hygiene outcomes, we found moderate-quality evidence in favour of the champion intervention. It is perhaps not surprising that there is unclear evidence on resident clinical outcomes, since we would only anticipate change on these outcomes if the implementation intervention was successful at changing staff behaviour to provide the recommended guideline-based care. For eight of 10 studies, this information was not available and thus, it is unclear why resident outcomes remained unchanged. In the two studies that did measure guideline adherence and resident outcomes, the champion intervention had a positive effect on improving both staff adherence and residents’ clinical health outcomes [6, 39].

### Findings in relation to other research

A recent integrative review [48] examining the role of the champion in supporting the implementation of evidence-based interventions into practice also found that champions, as a vehicle for implementation, exerted a positive influence on adherence to guidelines, recommendations and other relevant outcomes. Of the four randomised studies considered in this review, three were in areas we did not cover in the present review (neonatal units, schools, acute care hospital wards). The fourth was McCabe et al. [38], which is included in our review. Similar to our review, each of these studies found the presence of a champion led to a favourable outcome [48]. Thus, it would seem that our findings, although limited by the number of studies in this comparison, are in line with findings in other settings.

While our dataset is not sufficient to carry out post hoc analyses to explore which types of guidelines or interventions might benefit most from using a champion to boost implementation, we can draw on behaviour change theory and related evidence to infer how champions may be most effective. Perhaps the most comprehensive resources for designing theory-informed behaviour change interventions were produced by Michie and colleagues [49–51]. These include the Theoretical Domains Framework (TDF) the Behaviour Change Technique (BCT) Taxonomy, and the theory and techniques tool) [49–51]. The TDF framework is a synthesis of 33 different theories and includes 14 domains that represent the main drivers of behaviour change (e.g. knowledge, skills, social influences) [49]. The BCT Taxonomy provides a list of 93 techniques that can be used to change behaviour; these form the active components of an intervention (e.g. instruction on how to perform the behaviour, modelling, goal-setting, social support) [50]. The theory and techniques tool indicates which BCTs have been shown to be effective for each of the 14 TDF domains [51]. From a theoretical perspective, if we have an understanding of the TDF domains that are relevant to the implementation of a particular guideline or intervention, as well as an understanding of which domains are likely to be impacted by a champion, we can, at least conceptually, understand whether a champion is likely to be a useful implementation strategy to support adherence to that guideline or intervention.

We used the resources developed by Michie et al. [50] to first identify the behaviour change techniques at work in a typical champion-based intervention (see Additional file 5 for a list of common champion roles and responsibilities, the implicated BCTs and the TDF domains to which they relate). Using the theory and techniques tool, we then determined which TDF domains were linked with those techniques. The BCTs identified amongst champion roles and responsibilities were most commonly related to 4 key TDF domains that would determine behaviour change:

1. Beliefs about capabilities (e.g. verbal persuasion about capability which could be involved in a mentoring role)
2. Knowledge (e.g. provision of information commonly delivered through education sessions to staff members)
3. Beliefs about consequences (e.g. salience of consequences which would occur when delivering staff education)
4. Social influences (e.g. social support which would be a part of communication and building relationships with staff).

Therefore, from a theoretical perspective, the champions in these studies (with roles as described in Additional file 5) would be best placed to support teh implementation of and adherence to guidelines in which there were would likely be issues with, for example, lack of confidence to follow the guidelines, knowledge, social support or problems related to incorrect or unhelpful beliefs about the outcomes of following the guideline. For guidelines that may have other obstacles for implementation such as the ability of the healthcare professionals to retain required information (memory, attention and decision processes) the champion strategy, as commonly used in the literature and as enacted in the included studies in this review, may not yield the desired impacts on guideline adherence.

### Strengths and limitations of the review

Only RCTs and cluster RCTs were included in this review. Other study designs, more susceptible to bias, were excluded. To avoid selection bias, all references were screened, data-extracted and RoB assessed by two reviewers. There is also the possibility of publication bias, where studies reporting a null effect of the intervention are not submitted for publication, or if submitted are not accepted for publication. While we did try to mitigate this by searching for grey literature, we were unable to assess the possible extent of publication bias due to the heterogeneous nature of the interventions.

### Limitations of included studies

It is important to note that while the general duties of the champion were reported in most studies, many aspects of the interventions were not reported in sufficient detail to allow for replication or a more comprehensive understanding of intervention procedures for choosing or training the champions. For example, none of the included studies indicated how the champion was chosen or described the training provided to the champion (beyond the number of hours of training provided). While most studies provided the general role of the champion, the day to day procedures of how they enacted their role was missing, limiting our understanding of what the champions actually did. Most studies included small sample sizes and few actually measured adherence to guidelines which is the main aim of any implementation intervention, limiting the ability to determine its effectiveness. Moreover, important outcomes such as costing or resource use were rarely assessed so there was little information available for those who would wish to replicate or adopt the intervention.

### Implications for clinical practice

While this review found some evidence to support the use of champions in multicomponent interventions to implement guidelines, at this time, the evidence is not strong enough to recommend their widespread use without further understanding their role and the impact on cost. For example, in each of these interventions, the champion held major responsibilities and extra duties which appear consistent with the definitions of champions in the wider LTC literature [17]. However, we have to consider the impact of any additional duties a champion role may have and what resource implications that may have. Without knowing the exact benefit of the champion portion of the intervention it is premature to suggest that this is a reliable implementation strategy. Moreover, the varied nature of the champion role across studies in terms of the scope of their duties also makes it hard to recommend a champion since we do not know which duties are most effective for change.

### Future research

Future research should focus on designing studies with larger sample sizes and more robust methods to isolate the effects of the champion. For example, given that the use of champions may have resource implications, future studies should consider evaluating the additive clinical and cost-effectiveness of a champion to ascertain any added value for LTC homes. Additionally, investigators using cluster RCTs are popular in LTC settings should ensure they adjust for clustering in their analysis as per guidelines by Campbell et al. [52] to reduce risk of misleading results [52]. Of particular importance is the assessment of staff adherence in combination with resident clinical outcomes which was missing from the majority of studies. Finally, investigators should report (a) interventions in line with TiDier guidelines [28] to allow us to better understand the exact role of the champion and replicate or scale-up the intervention and (b) methodological components in line with the CONSORT statement to enable accurate risk of bias assessment.

## Conclusions

The findings suggest that champions may improve staff adherence to evidence-based guidelines in LTC homes. These results align with evidence from champion interventions in other settings. However, the certainty around these findings remains low due to methodological issues and poor reporting of the included studies. It is premature to recommend the widespread use of champions to improve uptake of guideline-based care in LTC homes without further study of the champion(s)’ role and its impact on resources and cost.

## Supplementary Information

**Additional file 1.** PRISMA checklist.

**Additional file 2.** Example of search strategy (MEDLINE).

**Additional file 3.** Summaries of included studies.

**Additional file 4.** Risk of bias table.

**Additional file 5.** Champion roles linked with relevant behavior change techniques and associated theoretical domains.

## Data Availability

All data generated or analysed during this study are included in this published article [and its supplementary information files].
