# Supplementary material for: Champions for improved adherence to guidelines in long-term care homes: a systematic review
Source: Implement Sci Commun. 2021 Aug 3;2:85. doi: 10.1186/s43058-021-00185-y (PMC8330034; doi:10.1186/s43058-021-00185-y)
Supplement: Supplementary file 2 — Additional file 2. Example of search strategy (MEDLINE). [file 43058_2021_185_MOESM2_ESM.docx]

Additional file 2. Example of search strategy (MEDLINE)

1. (elder$ or geriatric or geriatrics or old or older or aged or senior or seniors or veteran$).mp. [mp=title, abstract, original title, name of substance word, subject heading word, keyword heading word, protocol supplementary concept, rare disease supplementary concept, unique identifier]
2. (champion or champions or coordinator$ or Facilitator$ or aide or aides or carer or “care staff ” or “healthcare worker” or “certified nursing assistant” or ausculation).mp. [mp=title, abstract, original title, name of substance word, subject heading word, keyword heading word, protocol supplementary concept, rare disease supplementary concept, unique identifier]
3. 1 and 2
4. (“long term” or “long-term” or “care home$” or “nursing home$” or “care of the elderly” or “continuing care” or “sub acute” or “subacute” or “sub-acute” or “residential care”).mp. [mp=title, abstract, original title, name of substance word, subject heading word, keyword heading word, protocol supplementary concept, rare disease supplementary concept, unique identifier]
5. 1 and 2 and 4
6. (“long term” or “long-term” or “care home$” or “nursing home$” or “continuing care” or “sub acute” or “subacute” or “sub-acute” or “residential care”).mp. [mp=title, abstract, original title, name of substance word, subject heading word, keyword heading word, protocol supplementary concept, rare disease supplementary concept, unique identifier]
7. 1 and 2 and 6
8. (champion or champions or coordinator$ or facilitator or aide or aides or “train the trainer”).mp. [mp=title, abstract, original title, name of substance word, subject heading word, keyword heading word, protocol supplementary concept, rare disease supplementary concept, unique identifier]
9. 1 and 6 and 8
10. (champion or champions or coordinator$ or facilitator or aide or aides).mp. [mp=title, abstract, original title, name of substance word, subject heading word, keyword heading word, protocol supplementary concept, rare disease supplementary concept, unique identifier]
11. 1 and 6 and 10
12. 1 and 10
