# Supplementary material for: Champions for improved adherence to guidelines in long-term care homes: a systematic review
Source: Implement Sci Commun. 2021 Aug 3;2:85. doi: 10.1186/s43058-021-00185-y (PMC8330034; doi:10.1186/s43058-021-00185-y)
Supplement: Supplementary file 3 — Additional file 3. Summaries of included studies. [file 43058_2021_185_MOESM3_ESM.docx]

Additional file 3 - Summary of studies

| **Study** | **Characteristics** |
| --- | --- |
| Beekman 2013 | **Evidence-based recommendation:** Pressure-ulcer prevention guidelines  **Aim:** To decrease the occurrence of pressure-ulcers among residents in LTC homes  **Design:** cluster RCT  **Sample size:** Baseline: 11 Clusters (wards) (I=6, C=5) from 4 LTC homes. 118 Staff (I=65, C=53). 464 Residents (I=225, C=239)  Follow-up (120 days): 11 clusters, 118 staff, 464 residents (no loss to follow-up was reported)  **Intervention:** The intervention included training to use the decision support system by the research team and subsequent training on implementation and daily reminders about using the system. The champion worked with the research team to identify recommendations and implementation strategies that would be feasible within the unit and were involved in delivering education to staff, participating in monthly progress monitoring and problem solving with research team. However, the intervention also included a variety of other strategies not delivered by the champion including poster and pocket reminders and monthly monitoring and feedback on performance by the research team. Thus, the champion’s level of involvement in intervention delivery was judged to be moderate.  **Role of the champion:** moderate  **Staff adherence:** Staff adherence to the prevention protocol was assessed using an algorithm was based on a pilot study by Vanderwee et al. (2007) and included the combination of (1) the use of a pressure redistributing surface (mattress or cushion), (2) the frequency of repositioning, and (3) offloading of the heels in residents at risk for pressure ulcers (Vanderwee et al., 2007). Prevention was defined as fully adequate if all preventive measures were applied. Prevention was defined as partly adequate if not all required preventive measures were applied. Prevention was defined as not adequate if no prevention was applied. Note it did not say how many staff were assessed for this outcome  **Resident outcomes:** presence of pressure ulcers were measured by skin observation and categorized according to the 2009 EPUAP/NPUAP classification system.  **Analysis issues:** yes, the analysis did not adjust for clustering. |
| Chami | **Evidence-based recommendation:** consensus recommendations were established for infection control in elderly people living in NHs.  **Aim:** To reduce the infection rates of commonly encountered infections.  **Design:** Cluster RCT  **Sample size:** Baseline: 50 Clusters (LTC homes) staff not reported, 4515 residents. Follow-up (5 months): 47 clusters, 4515 residents  **Intervention:** The intervention was primarily education-based focusing on infection prevention strategies (hand hygiene, disinfecting, oral health, malnutrition and dehydration). It included posters as reminders as well as additional tools such as hand sanitizer, disinfectant sprays and toothbrushes. The champion was trained to deliver all the education training to staff thus playing a major role in the intervention. The research team also assessed staff knowledge and compliance with a self-report monthly survey.  **Role of the champion:** major  **Staff adherence:** not formally assessed.  **Resident outcomes:** total infection rate (urinary, pneumonia, bronchitis and/or tracheobronchitis, influenza-like illness, otitis, sinusitis, rhinitis, and gastrointestinal infections) classified either as definite or probable. It was measured using a standardized checklist completed by the research team using medical case notes (written diagnosis) and biologic and/or radiologic confirmation if available.  **Analysis issues:** No, the analysis adjusted for clustering. |
| DeVisschere 2012 | **Evidence-based recommendation:** To improve the oral health of residents by maintaining daily oral hygiene care by removing the bacterial plaque on teeth and dentures according to the Dutch guideline ‘Oral health care in (residential) care homes for elderly people’.  **Aim:** to improve the daily oral hygiene of residents  **Design:** Cluster RCT  **Sample Size:** Baseline: 12 clusters (LTC homes), 760 staff, 373 residents. Follow-up: 12 clusters, (unclear about residents but it seems like 295 were assessed for dental and denture plaque, note this likely includes some overlap as some people may have been assessed in both groups, but this is all that was reported)  **Intervention:** The intervention consisted of an initial education session by the research team followed by ongoing education and training delivered by the champion on how to undertake proper oral hygiene using the oral products provided by the research team.  **Role of the champion:** major  **Staff adherence:** not assessed  **Resident outcomes:** The study measured dental and denture plaque using the Silness and Loe validated plaque index and the Augsburger and Elahi Methylene Blue denture plaque disclosing solution respectively  **Analysis issues:** No, the study adjusted for clustering. |
| Gaskill | **Evidence-based recommendation:** To prevent malnutrition, diets should include considerations such as fortified food, supplements and meal planning; appropriate eating assistance during mealtimes; modification of the environment to create an atmosphere conducive to eating; and assessment and monitoring of weight history and eating behavior.  **Aim:** To reduce malnutrition among LTC home residents  **Design:** cluster RCT  **Sample Size: Baseline:** 8 clusters (LTC homes), staff number not reported, 352 residents. Follow-up (6 months): 8 clusters, staff number not reported, 279 residents.  **Intervention:** The nutrition intervention was education-based on how to assess for risk of malnutrition with poster reminders outlining the malnutrition risk assessment and action strategies. The intervention used a train-the-trainer approach and trained a site champion on how to educate other nursing and kitchen staff on malnutrition risks and action strategies including how to modify menus and other behaviour change strategies. In this way the champion played a main role in the intervention.  **Role of the champion** (minor, moderate, major): major  **Staff Adherence**: not assessed  **Resident Outcomes:** The malnutrition prevalence SGA consists of the resident’s medical history (weight loss, dietary intake, gastrointestinal symptoms and functional capacity) and a physical assessment of subcutaneous fat, muscle wasting and edema. Residents were allocated a SGA rating and categorized as well nourished (SGA A), moderately malnourished or suspected to be malnourished (SGA B) or severely malnourished (SGA C). Scores were dichotomized as well-nourished or moderate-severely malnourished.  **Analysis issues**: unclear, the authors state that they have adjusted for clustering in the design but did not state how and the final analysis was adjusted only for age, gender, pretest nutritional status and level of care but not the cluster level itself. |
| Livingston 2019 | **Evidence-based recommendation:** to provide care to reduce agitated behaviour in people with dementia  **Aim:** to train staff in how to assess a patient with dementia and to understand agitation and to make a plan that would include pleasant events and environmental changes to reduce agitated behaviour.  **Design:** Cluster RCT  **Sample Size:** Baseline: 20 clusters (LTC home), 492 staff, 404 residents. Follow-up (8 months): 20 clusters, 492 staff, 318 residents.  **Intervention:** the intervention focused on teaching staff strategies to manage agitation, the main components included 6 education sessions delivered by the research team, development of actions plans for each care home including environmental and practical strategies to manage agitation followed by monthly supervision by the research team; the champions had a very minor role in this intervention and were primarily responsible for helping to coordinate the education sessions (e.g. make sure there was a room booked and materials were available when needed) as well as remind staff about the intervention.  **Role of the champion:** minor  **Staff adherence:** not assessed  **Resident outcomes:** The study measured resident agitation using the 29-item Cohen-Mansfield Agitation Inventory completed by the primary caregivers for assessment. The study also assessed quality of life using the Dementia Specific QoL scale.  **Analysis issues:** No issues - the study adjusted for clustering. |
| MacEntee 2007 | **Evidence-based recommendation:** to provide oral hygiene (such as tooth examination and toothbrushing) for residents.  **Aim:** to improve the oral hygiene of residents by educating staff on how to provide this level of care according to best practices.  **Design:** Cluster RCT  **Sample Size:** Baseline: 14 clusters (LTC homes), 812 staff, 152 residents. Follow-up (3 months): 13 clusters, 113 residents.  **Intervention:** The intervention consisted of education and training delivered by the champion with no other strategies.  **Role of the champion:** major  **Staff adherence:** not assessed  **Resident outcomes:** This study measured oral debris and gingival inflammation using the Geriatric Simplified Debris Index (GDI-S).  **Analysis issues:** No issues, the study adjusted for clustering |
| McCabe 2013 | **Evidence-based recommendation:** to assess for depression amongst residents in LTC home to aid in early identification and appropriate treatment planning.  **Aim:** to education and train nursing staff in how to recognize and monitor depression.  **Design:** RCT with 3 groups  **Sample size:** Baseline: 107 Staff (Ia=34, Ib,=35, C=38). 216 Residents. Follow-up: no loss to follow-up was reported  **Intervention:** The first intervention was an educational training program on how to recognize depression as well as use multiple assessment tools for depression. The second intervention included the same educational training program but also included a staff champion who was responsible for using a new screening tool to screen residents for depression and for liaising with junior staff regarding the educational training program. The control group received no intervention.  **Role of the champion:** major  **Staff adherence:** Staff adherence was defined by how many staff in each group correctly identified residents who were depressed which was measured by concordance between staff diagnosis and diagnosis using the Statistical Manual for Mental Disorders (SCID).  **Resident outcomes:** presence of pressure ulcers were measured by skin observation and categorized according to the 2009 EPUAP/NPUAP classification system.  **Analysis issues:** This was not a cluster trial – the RCT appeared to follow an intention to treat analysis but it was not explicitly stated. |
| Resnick | **Evidence-based recommendation:** preventing functional decline in LTC homes  **Aim:** to provide function focused care to improve resident’s functional status in LTC homes.  **Design:** cluster RCT  **Sample Size:** Baseline: 4 Clusters (I=2, C=2). 96 Staff (I=50, C=46). 171 Residents (I=93, C=78)  Follow-up (12 month): 4 Clusters (I=2, C=2). 69 Staff (I=36, C=33). 101 Residents (I=54, C=47)  **Intervention:** The intervention involved a series of sequential components led by the research team: an environmental and policy assessment to recommend appropriate and affordable interventions to optimise function, 30 minutes of staff training, physical function goal setting for residents, and monitoring of performance over 12 months with one-to-one mentoring as needed. The champions played a major role in enacting parts of the intervention including developing the initial intervention options with the research team and ensuring the residents’ functional goals were written into the resident’s chart as well as providing ongoing education with the research team over the 12-month intervention.  **Role of the champion:** major  **Staff adherence**: Staff adherence to providing function focused care was assessed by observation of LTC home staff during care interactions using the Restorative Care Behavior Checklist. The observations lasted 30 minutes and nineteen care-related interactions were evaluated (e.g., bathing, dressing, ambulating).  **Resident outcomes:** Physical function was measured by the 10-item Barthel index which assesses 10 functional activities and is rated on a 0-100 scale. Shah et al have suggested that scores between 61-90 indicate moderate dependency. Adverse events (Falls, Deaths, or Injury) were also measured.  **Analysis**: unclear, although the methods state the analysis will be adjusted for clustering, the data reported appear to be unadjusted scores for each group with a significance test. |
| Siddiqi 2016 | **Evidence-based recommendation:** recommendations for delirium prevention and management  **Aim:** To reduce / prevent episodes of delirium  **Design:** a feasibility Cluster RCT  **Sample size:** Baseline: 14 clusters (LTC homes), staff number not reported, 215 residents. Follow-up (16 months): 14 clusters, staff number not reported, 160 residents  **Intervention:** The intervention included two main components of education and goal setting. It also used a train the trainer approach in which the research team trained champions at each site to provide ongoing education to new staff and lead the monthly goal setting sessions, which we have categorized as a major role for the champion.  **Role of the champion:** major  **Staff adherence:** not assessed  **Resident outcomes:** The study used the Delirium Rating Scale-Revised-98 for assessment which was completed by members of the research team. The study also assessed quality of life using the EQ-5D.  **Analysis issues:** yes, the study did not adjust for clustering in their analysis (note: this was a feasibility study and it did not report between adjusted or unadjusted group differences). |
| Van de ven 2013 | **Evidence-based recommendation:** Using person-centred care for dementia management  **Aim:** to implement the person-centred Dementia-care mapping intervention in long-term care homes to reduce patient agitation  **Design:** Cluster RCT  **Sample Size**: Baseline: 11 clusters (LTC homes), 382 staff, 434 residents. Follow-up (8 months): 11 clusters, 280 staff, 175 residents.  **Intervention:** the intervention focused on teaching staff dementia-care mapping skills which included developing action plans, the components included a brief overview to staff by the research team followed by training at least two champions at each site to be a dementia-care mapper who would then teach, observe and provide feedback to other staff at their site; in this way the champion played a major role in the intervention.  **Role of the champion:** major  **Staff adherence:** not assessed  **Resident outcomes:** The study measured resident agitation using the 29-item Cohen-Mansfield Agitation Inventory completed by the primary caregivers for assessment. The study also assessed quality of life using the EQ-5D.  **Analysis issues:** no issues, the study adjusted for clustering. |
| Van den Block 2020 | **Evidence-based recommendation**: providing palliative care in nursing homes.  **Aim:** to implement the Palliative Care for Older People (PACE) Steps to Success Program to provide palliative care in nursing homes  **Design:** Cluster RCT  Sample Size: Baseline: 78 clusters (LTC homes), 610 residents. Follow-up (12 months): 73 clusters, 913 residents.  **Intervention:** The intervention consisted of education and training delivered by the champion with no other strategies.  **Role of the champion:** major  **Staff adherence:** not assessed  **Resident outcomes:** This study measured comfort in the last week of life by staff using the End-of-Life in Dementia Scale Comfort Assessment while dying (EOLD-CAD) tool as well as satisfaction with care from a relative’s perspective using the End of-Life in Dementia–Satisfaction with Care tool.  **Analysis issues:** no issues, the study adjusted for clustering. |
| Van der Putten 2013 | **Evidence-based recommendation:** The “Oral health care Guideline for Older people in Long-term care Institutions (OGOLI)” was followed, which includes educating nursing staff about oral health care  **Aim:** To implement the OGOLI guidelines in long-term cares homes to reduce dental and denture plaque scores of residents  **Design:** Cluster RCT  **Sample Size:** Baseline: 12 clusters (LTC homes), Staff number not stated, 342 residents. Follow-up (6 months): 12 clusters, Staff number not stated, 232 residents were assessed for denture and dental plaque.  **Intervention:** This study used the same intervention as DeVisschere 2012. The intervention consisted of an initial education session by the research team followed by ongoing education and training delivered by the champion on how to undertake proper oral hygiene using the oral products provided by the research team.  **Role of the champion:** major  **Staff adherence:** not assessed  **Resident outcomes:** The study measured dental and denture plaque using the Silness and Loe validated plaque index and the Augsburger and Elahi Methylene Blue denture plaque disclosing solution respectively  **Analysis issues:** no issues, the study adjusted for clustering. |
