# Supplementary figures and images for: Champions for improved adherence to guidelines in long-term care homes: a systematic review
Source: Implement Sci Commun. 2021 Aug 3;2:85. doi: 10.1186/s43058-021-00185-y (PMC8330034; doi:10.1186/s43058-021-00185-y)

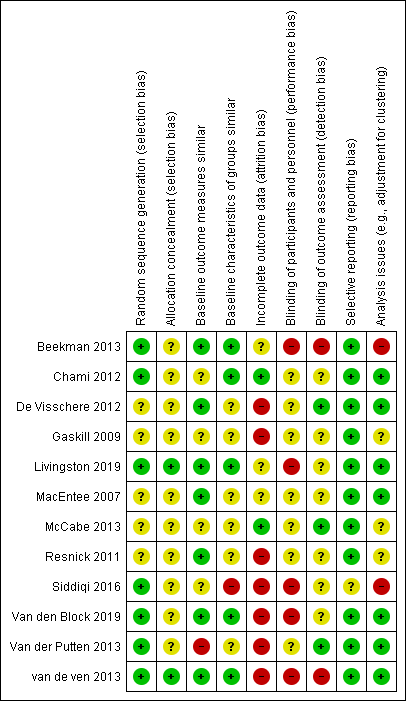


**Additional file 4.** Risk of bias

Supplement: Supplementary file 4 — Additional file 4. Risk of bias table. [file 43058_2021_185_MOESM4_ESM.docx]
