# Supplementary material for: Champions for improved adherence to guidelines in long-term care homes: a systematic review
Source: Implement Sci Commun. 2021 Aug 3;2:85. doi: 10.1186/s43058-021-00185-y (PMC8330034; doi:10.1186/s43058-021-00185-y)
Supplement: Supplementary file 5 — Additional file 5. Champion roles linked with relevant behavior change techniques and associated theoretical domains. [file 43058_2021_185_MOESM5_ESM.docx]

Additional file 5. Champion roles linked with relevant behavior changes techniques and associated theoretical domains.

| **Champion roles** | **BCTs** | **TDF Domains** |
| --- | --- | --- |
| Delivering staff education | 4.1 Instruction on how to perform the behavior | Knowledge, skills, beliefs about capabilities |
|  | 5.1 Information about health consequences | Knowledge, beliefs about consequences, intentions |
|  | 5.2 Salience of consequences | Beliefs about consequences |
|  | 5.3 Information about social and environmental consequences | Knowledge, beliefs about consequences |
| Problem-solving | 1.2 Problem-solving | Beliefs about capabilities, behavioral regulation |
| Coordination of intervention activities | 1.4 Action planning | Behavioral regulation* |
| Communication/building relationships with staff | 3.1 Social support (unspecified) | Social influences |
|  | 3.2 Social support (practical) | Environmental context and resources |
| Acting as a resource/mentor | 3.1 Social support (unspecified) | Social influences  Beliefs about capabilities |
|  | 3.2 Social support (practical) | Environmental context and resources, social influences |
|  | 15.1 Verbal persuasion about capability | Beliefs about capabilities |
|  | 15.3 Focus on past success | Beliefs about capabilities |
| Advocating/facilitating/leading/modelling practice change | 1.1 Goal-setting (behavior) | Intentions, goals |
|  | 1.4 Action planning | Behavioral regulation* |
|  | 6.1 Demonstration of the behavior | Beliefs about capabilities |
| Providing feedback | 2.2 Feedback on behavior | Knowledge*, reinforcement* |
| Monitoring progress | 2.1 Monitoring of behavior by others without feedback | Reinforcement*, social influences* |

Note: Domains marked with an * show only a weak association with the BCT.
